# Determination of Post-Harvest Biochemical Composition, Enzymatic Activities, and Oxidative Browning in 14 Apple Cultivars

**DOI:** 10.3390/foods10010186

**Published:** 2021-01-18

**Authors:** Sara Serra, Brendon Anthony, Francesca Boscolo Sesillo, Andrea Masia, Stefano Musacchi

**Affiliations:** 1 Department of Horticulture, Tree Fruit and Research Extension Center (TFREC), 1100 N. Western Avenue, Washington State University, Wenatchee, WA 98801, USA; sara.serra@wsu.edu (S.S.); brendon.anthony@wsu.edu (B.A.); 2 Department of Obstetrics, Gynecology, and Reproductive Sciences, University of California, San Diego, CA 92093, USA; fboscolo@health.ucsd.edu; 3 DipSA—Alma Mater Studiorum, University of Bologna, Viale Fanin 46, 40127 Bologna, Italy; andreamasia48@gmail.com

**Keywords:** antioxidants, polyphenol oxidase (PPO) activity, nutraceutical properties, polyphenols

## Abstract

Phenolic compounds in fruit provide human health benefits, and they contribute to color, taste, and the preservation of post-harvest fruit quality. Phenolic compounds also serve as modifiers of enzymatic activity, whether inhibition or stimulation. Polyphenol oxidases (PPO) and peroxidases (POD) use phenolic compounds as substrates in oxidative browning. Apple browning leads to flesh color, taste, texture, and flavor degradation, representing a drawback for the variety and its’ market appraisal. This study was conducted to investigate the process of browning in 14 apple cultivars throughout post-harvest at three-time points: immediately (T0), one hour (T1), and 24 h (T2) after apples were cut in half. Color parameters L* (lightness), a* (red/green), b* (yellow/blue) were measured, and chroma (ΔC*) and color (ΔE) were calculated to quantify differences between T0₋T1 and T1₋T2 on the fruit surface. Enzymatic activity (PPO, POD) and phenolic composition were also quantified for each cultivar. ‘Granny Smith’ and ‘Cripps Pink’ browned minimally. In contrast, ‘Fiesta’ and ‘Mondial Gala’ browned severely, reporting high enzymatic activity and quantified phenolic concentration (QPC). Phenolic compound polymerization appears to play a significant role in enzymatic inhibition. ‘Topaz’ does not fit the high QPC, PPO, and browning formula, suggesting alternative pathways that contribute to apple browning.

## 1. Introduction

In recent years, the attention of breeders, producers, and consumers alike have shifted towards ‘functional foods’, such as fruits high in biologically active phenolic compounds that are beneficial for human health [1,2,3]. Phenolic compounds are produced by plants and are involved in determining color and taste in fruit [4]. They also act as natural antioxidants, binding to molecular oxygen, which potentially limits damage to cells and tissues [5,6]. Higher consumption of fruits, which are a rich source of phenolic compounds, has been related to reduced risks of chronic and degenerative diseases [7]. Phenolics have also shown anti-cancer and anti-aging properties, helping prevent cardiovascular diseases and cataracts [8,9,10].

Beyond their health benefits, phenolic compounds are involved in the genetically-regulated ripening process in climacteric fruit, particularly the physiological and biochemical pathways that involve flesh softening, pigment synthesis, and the production of volatile and aromatic compounds [11]. These secondary metabolites and their metabolic processes can affect fruit shelf life, as well as sensorial and organoleptic qualities in fruit (e.g., browning, bitterness, and astringency) [2,12,13]. Phenolic compounds act as substrates for enzymatic activities that can turn fruit tissues “brown” during post-harvest handling, processing, and manipulation [14]. This phenomenon, known as enzymatic browning, leads to color, taste, texture, and flavor deterioration of the fruit, causing a reduction in the overall quality and value [13]. Enzymatic browning is a critical issue with apples, especially in the processing industry, which, after sulfites were banned, needed anti-browning agents for sliced apples [15,16].

Polyphenol oxidase (PPO; EC 1.10.3.1) is the most prevalent enzyme involved in the apple flesh browning process [14]. Generally, in plant cells, PPO is found in the plastids, while phenolic compounds are located in the vacuoles [13]. However, when damage occurs to the plant tissue (e.g., an apple is sliced or bruised during handling), the plastids and vacuoles are ruptured, allowing contact between the phenolic compounds (substrate) and PPO (enzyme) [13]. When PPO and phenolic compounds meet in the presence of O_2_, the enzyme catalyzes the oxidation of phenols to quinones, which subsequently polymerize and generate brown and insoluble pigments known as melanin [14,17,18]. The fully sequenced genome of ‘Golden Delicious’ revealed that PPO is encoded across 10 genes distributed among three chromosomes [19,20]. This has led to the development of genetically engineered non-browning apples, known as the ‘Arctic’ series, which have the genes responsible for PPO expression disrupted [21]. Overall, enzymatic activity and phenolic concentrations appear to be cultivar-specific, as do their degree of browning in apples [4].

In addition to PPO, the enzyme peroxidase (POD; EC 1.11.1.7) is also involved in the loss of color and the modification of taste, flavor, and nutritional properties that compromise fruit quality [22,23]. PODs belong to the family of oxidoreductases, which in the presence of H₂O₂, catalyze tyrosine residues’ oxidation and, again, induce melanin [24,25]. This enzyme is also involved in the last steps of lignin biosynthesis and defense mechanisms against pathogens and microorganisms [26,27,28].

Apples are one of the most produced and consumed fruits globally and contain high levels of phenolic compounds [7]. Phenolic profiles of apple are well-characterized, and the five major phenolic groups found in most cultivars include: hydroxycinnamic acids, flavan-3-ols and procyanidins, anthocyanins, flavonols, and dihydrochalcones [7,29,30,31,32]. Generally, a correlation exists between the level of PPO activity, total phenolic concentration (TPC), and enzymatic browning, although this relationship’s strength appears to be cultivar specific [4]. Furthermore, the phenolic compound composition may vary depending on the fruit’s maturity status and climatic/environmental conditions [4,26,33]. Phenolics serve as translators for physiological adjustments, connecting the environment with the fruit’s biology [34]. However, more than this, phenolic compounds act as strong modifiers of enzymatic activity, both in respect to inhibition and stimulation, as documented in apple bud initiation [34,35]. For example, polymerized procyanidins have demonstrated an inhibitory effect on PPO activity [35]. Therefore, in each apple cultivar, the distinct phenolic composition and the activity of various enzymes are critical to understanding, as they may each influence the potential for browning.

The objective of this study aimed to identify and quantify the total and specific phenolic compounds in an array of 14 apple cultivars at three-time points post-slicing (immediately after, 1 h, and 24 h after the cut), along with their degree of browning (i.e., color variation) and enzymatic activity (e.g., PPO, POD, and indole-3-acetic acid oxidase (IAAox).

## 2. Materials and Methods

### 2.1. Apple Cultivars and Sampling

In 2005, 14 apple cultivars were obtained from a germplasm collection located in the experimental station of the University of Bologna (Cadriano, Bologna, Italy). Selected cultivars were: ‘Braeburn’, ‘Cripps Pink’, ‘Delorina’, ‘Durello’, ‘Fiesta’, ‘Florina’, Nagafu 12—a strain of ‘Fuji’, ‘Gloster’, ‘Golden Delicious’, ‘Granny Smith’, ‘Jonathan’, ‘Mondial Gala’, ‘Red Rome’, and ‘Topaz’. Apples were harvested at the commercial ripening stage and subsequently stored in a cold room held at 0−2 °C. Starch-iodine tests were conducted to assess the range of maturity across the varieties evaluated. Starch degradation was ranked on a scale from 1 (unripe) to 10 (ripe), with average levels in this experiment ranging from 6–10 to ensure a minimum of commercial harvest maturity.

Five apples were selected for each cultivar and cut cross-sectionally, following each apple’s equatorial line perpendicular to the imaginary axis connecting stem and calyx. This allowed the evaluation of the enzymatic browning process. The apple flesh color changes were measured on the apple cut surface by a Colorimeter (CR-300, Konica-Minolta Co. Ltd., Osaka, Japan) set as a tri-stimulus mode. Three different parameters described the flesh color: L* (lightness), a* (greenness-redness), and b* (yellowness-blueness). These colorimetric parameters (L*, a*, b*) were be used to calculate the color difference (ΔE) following the equation ΔE = [(ΔL)^2^ + (Δa)^2^ + (Δb)^2^]^1/2^ and the chroma difference (ΔC) as ΔC = [(Δa)^2^ + (Δb)^2^]^1/2^ [36], as they express color according to human perception [37]. Three color measurements were performed on the bottom half of each apple at three-time points: immediately after apple cutting (T0), 1 h after the cut (T1), and 24 h after the cut (T2). Color coordinates were captured at these three-time points (T0, T1, T2) to determine the degree of color change (ΔE) and chroma change (ΔC*) across two periods of time: between the initial slice and one-hour after slicing [ΔE1, ΔC*1 (T1–T0)], and 24 h after that [ΔE2, ΔC*2 (T2–T1)]. The T2 measurement was established according to Kim et al. [38], who showed maximum PPO activity in ‘Fuji’ after this amount of time. All apples (bottom hemisphere) were peeled immediately after the T0 measurements.

Six flesh aliquots (three for polyphenols and three for enzymatic analyses) were collected for each cultivar at T0, T1, and T2. Each aliquot ranged from 10 to 15 g for phenolic determination and from 1.5 to 2.0 g for enzymatic assays [39,40]. Each aliquot was made of collected flesh (i.e., mesocarp) material from the equatorial region from a pool of pieces of the three selected apples per each time point. All samples were flash-frozen in liquid nitrogen and stored at −80 °C for a few weeks until used for further analyses.

### 2.2. Extraction and Enzymatic Activity of POD and PPO

An aliquot of less than 2 g of frozen apple flesh, sampled from the equatorial region of the central portion of the mesocarp, was pulverized with liquid nitrogen to obtain a powder for enzymatic analysis. The frozen powder was added to 10 mL of an ice-chilled buffer made 200 mM, phosphate buffer (pH 7.0), 5 mM Na_2_EDTA (disodium ethylenediaminetetraacetic acid), and 0.1 g of PVPP (polyvinyl polypyrrolidone) (Sigma-Aldrich, St. Louis, MO, USA). The samples were centrifuged at 10,000 rcf for 30 min at 4 °C (Beckman Coulter, Avanti J20XP, Palo Alto, CA, USA), and the supernatant was collected and used for the activity analysis following the method described in Masia et al. [41]. The POD activity (EC 1.11.1.7) was determined spectroscopically using pyrogallol as a substrate for the enzymatic reaction, and the absorbance was measured at 430 nm after 10 min incubation at 20 °C [42]. The PPO activity (EC 1.10.3.1) was evaluated according to Cañal et al. [43] at 420 nm after 15 min incubation at 30 °C. Enzymatic activity values were expressed as a units (u) of enzyme per gram of apple flesh fresh weight (FW) [43]. The IAAox activity (E.C. 1.11.1.8) was assessed by determining absorbances at two wavelengths, 247 nm and 254 nm [41].

### 2.3. Extraction of Apple Phenolics and Quantification by High-Performance Liquid Chromatography (HPLC)

An aliquot of 15 g of frozen apple flesh chunks sampled from the equatorial region of the central portion of the mesocarp was homogenized in 20 mL of 70% chilled acetone (extraction solution, Sigma-Aldrich, St Louis, MO, USA) in a glass jar adapted for the Osterizer Sunbeam blender (model 4153-50, Oster, Boca Raton, FL, USA). The blender’s initial speed was set at ‘2’ to promote the breakdown of sizeable frozen apple pieces. After ca. 30 s, the speed was increased to ‘10’ and the sample was homogenized for 60 s. The homogenized sample and an appropriate volume of the extraction solution were added to ultracentrifuge tubes and rotated at 10,000 rcf for 20 min at 4 °C (Beckman Coulter, Avanti J20XP, Palo Alto, CA, USA). The supernatant was collected, and the centrifugation steps were repeated twice more for 10 min each. The supernatants collected from the three centrifugation steps were mixed together. A 100 mL final volume was obtained by adding the extraction solution to the supernatant. All samples were filtered by syringe with 0.45 µm polytetrafluoroethylene (PTFE) filters (Sigma-Aldrich, St Louis, MO, USA), and a further speed vacuum rotation was performed to eliminate the organic components (acetone) and to obtain pellets. Pellets were freeze-dried and stored at −20 °C for a few days until further analyses.

Samples for HPLC analysis were prepared as follows: each freeze-dried pellet was re-suspended in 500 µL of methanol and 500 µL of Milli-Q water and subsequently filtered through 0.22 µm polyvinylidene difluoride (PVDF) filters (Sigma-Aldrich, St Louis, MO, USA), into vials. The analysis was conducted in an HPLC apparatus made of a Waters 1525 binary pump, Waters Inline degasser AF, photodiode detector (PDA, Waters 2996), auto-sampler (Waters 717 plus), and Waters Atlantis™ dC18 (5 µm, 4.6 mm × 250 mm) column (Waters Corporation, Yvelines, France). Two mobile phase solutions were adopted for all the samples and corresponded to 1% HCOOH + 0.5% MeOH (solution A); and 100% CH_3_CN (solution B) with 1 mL as total flux per minute. The following HPLC running settings were adopted for the two solutions: 0–11 min 9% B, 11–13 min 15% B, 13–20 min 17% B, 20–37 min 60% B, 37–38.5 min 100% B, 38.5–39.5 min 9% B. The injection volume equaled 20 µL, and the total running time for each sample was 39.50 min. Five minutes of reconditioning time were allowed between samples to stabilize the column. Empower™ 2 software (Waters Corporation, Yvelines, France) was employed to analyze the results.

The detection of compounds was carried out at 280 nm for flavan-3-ols (e.g., catechin and epicatechin) and dihydrochalcones (e.g., phloridzin) and at 320 nm for hydroxycinnamic acids (e.g., chlorogenic acid and *p*-coumaric) and both retention time and ultraviolet (UV) spectrum were evaluated in the identification of the compound. Quantification was performed with the external standard method with the standards calibration curves (four different concentrations to build each curve: 25, 50, 100, and 200 μg mL^−1^). The reliability of the quantification method was assessed by the *R*^2^ value from the standards calibration curves. The chosen standards were catechin, epicatechin (for flavan-3-ols), chlorogenic acid, and *p*-coumaric acid (for hydroxycinnamic acids, Sigma-Aldrich, St. Louis, MO, USA), and phloridzin (for dihydrochalcones or bicyclic flavonoids, Extrasynthese, Lyon, France) since they are highly represented in apple flesh [29]. Proanthocyanidins were quantified based on the catechin standard. The non-identified peaks were assigned to a specific class according to their UV spectrum, and the comparison was made to standards with associated absorbance values. Each compound’s final concentration was presented as mg per gram of fresh apple weight (FW). The compounds detected were then summed and presented as quantified phenolic concentration (QPC).

### 2.4. Statistical Analyses

Flesh color change (i.e., browning), phenolic compound concentration, and enzymatic activity were analyzed for cultivar and times of sampling. The analysis of variance was performed using Proc GLM in Statistical Analysis System software (SAS Unversity Edition 2; SAS Institute Inc., Cary, NC, USA). The significance of the method was for *p* < 0.05 with the type III sums of squares test. Multiple comparison tests were assessed with Tukey honest significant difference (HSD) or Student Newman-Keuls (SNK) for post-hoc mean separation. Figures and tables reported different letters associated with the means when the model’s significance was at *p* < 0.05. Apple phenotypic and biochemical characteristics were also investigated via principal component analysis (PCA) performed in JMP (SAS Institute Inc., Cary, NC, USA). Data imagining was conducted in Prism v8.2.1 for Windows OS (Graph Pad Inc., San Diego, CA, USA) and presented in the figures as means ± standard error. When error bars are not displayed in figures, Tukey’s least significant difference (LSD) is displayed.

## 3. Results and Discussion

### 3.1. Apple Flesh Color Changes with Respect to Time Post-Slicing and Cultivar

**Figure 1:**
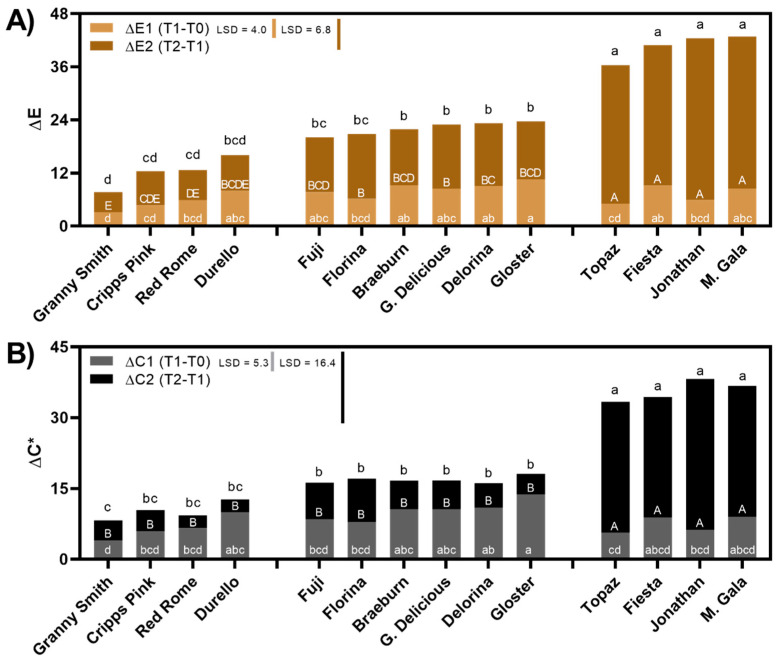
(**A**) Color difference between each time point as ΔE between T1 (1 h from cut) and T0 (cut) and between T2 (24 h from cut) and T1 (1 h from cut) across cultivars. Means are displayed for each time with the sum of ΔE from T2–T0 (ΔE) stacked in one column. Different black letters above the columns indicate differences according to Tukey post-hoc mean comparison at a *p* < 0.05 for ΔE (T2–T0) as the total color difference from T0 to T2. LSD determined by Tukey for ΔE1 and ΔE2 are displayed in the top left corner. Letters (in white) inside the columns denote differences in means at ΔE1 (lower-case inside light brown column) and ΔE2 (upper-case inside darker brown columns). (**B**) Chroma difference between each time point as ΔC* (chroma) between T1–T0 and T2–T1 across cultivars. Means are displayed for each time with the sum of ΔC* from T2–T0 (Δ*C*3) stacked in one column. Different letters above the column indicate differences according to Tukey post-hoc mean comparison at a *p* < 0.05 for ΔC* (T2–T0). LSD determined by Tukey for Δ*C*1 and Δ*C*2 are displayed in the top left corner. Letters (in white) inside the columns denote differences in means at Δ*C*1 (lower-case inside gray columns) as 1 h after cut and Δ*C*2 (upper-case inside black columns) as color change in the time between 1 h and 24 h.

Defined by CIE standards, L* (lightness), a* (red-green), and b* (yellow-blue) coordinates were captured to calculate color (E), along with the chroma (C*), which expresses the saturation (brighter or duller) of that color [36]. Both the color and chroma changes across time points (T0, T1, T2) are displayed by cultivar in Figure 1A,B. The total sum of color and chroma change from slice initiation to 24 h later (ΔE, ΔC*) is expressed as the total column for each cultivar (Figure 1A,B).

Due to different degrees of changing flesh coloration, or browning severity, the cultivars were sorted into three groups based on statistical separation: minimal browning, moderate browning, and severe browning (Figure 1). The degree of browning was determined by the total change in color (ΔE) (Figure 1A). The varieties whose pulp “browned” the most overall included: ‘Topaz’, ‘Fiesta’, ‘Jonathan’, and ‘Mondial Gala’, while the varieties that browned the least included ‘Granny Smith’, ‘Cripps Pink’, ‘Red Rome’, and ‘Durello’ (Figure 1A). ‘Topaz’ did not brown substantially across ΔE1, one hour after it was cut, but demonstrated a significant shift in color after 24 h (ΔE2) (Figure 1A). A similar trend was noted for ‘Jonathan’, which demonstrated minimal color variation in ΔE1, but incurred heavy browning post-24 h (Figure 1A). Previous literature confirms the moderate browning detected in ‘Florina’ and ‘Golden Delicious’ after one hour and in ‘Fuji’ after 24 h [4,44]. Additionally, ‘Topaz’ demonstrated moderate browning after one hour in Persic et al. [44], which confirms our T1 results (Figure 1A). However, ‘Topaz’ seems to brown excessively in the period of time following 1 h, in the present study, leading to severe browning by T2 (Figure 1A). In contrast to our results on ‘Granny Smith’ (Figure 1), Persic et al. [44] noted exceptional browning in this variety and attributed this to its high TPC levels at harvest. Although browning appears to be cultivar specific, other preharvest factors such as fruit maturity, age of the tree, geographic conditions, and seasonal variation can all impact the phenolic concentration and browning potential of a fruit. This could represent an explanation of differences in TPC, PPO, and browning in individual cultivars behaving differently from study to study [4,45,46]. For example, when assessing the maturity of the varieties evaluated via the starch-iodine test at T0, both ‘Granny Smith’ and ‘Red Rome’ were significantly less ripe than the other varieties (data not shown). Therefore, in contrast to the results in Persic et al. [44], ‘Granny Smith’s lack of browning may result from its less ripe maturity compared to the other cultivars (data not shown). However, ‘Cripps Pink’ and ‘Durello’, which also browned minimally, were not significantly different in respect to maturity than the other varieties (data not shown). This underscores that other factors beyond maturity must play a role in the oxidative browning potential of a variety.

**Figure 2:**
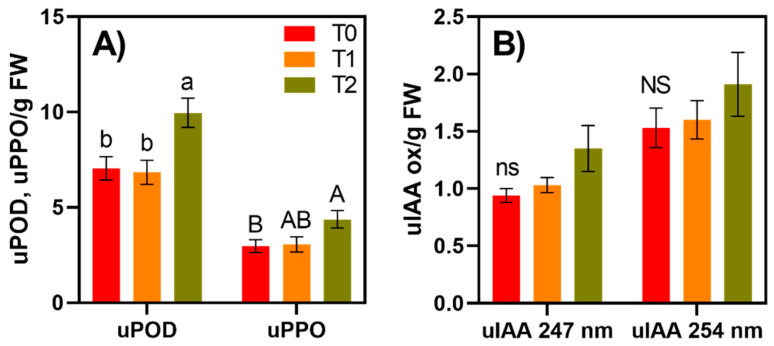
Change in enzymatic activity by time (T0 = immediately after apple cut, T1 = 1 h after cut, T2 = 24 h after cut): (**A**) = peroxidase units (uPOD) and polyphenol oxidase units (uPPO); (**B**) = indole-3-acetic acid oxidase units/g FW (uIAAox) over time. Means displayed are averages from all 14 cultivars. Means ± standard error displayed. Different letters above bars indicate differences according to Tukey post-hoc mean comparison at a *p* < 0.05. NS (non-significant difference) for IAA ox at either absorbance across time points. No significant interactions were detected by cultivar x time for enzymatic activities when assessed by ANOVA at *p* < 0.05.

Color changes were significant between T1 and T2, whereas chroma differences were more pronounced between T0 and T1, except for the severe browning cultivars (Figure 1A,B). Previous literature only demonstrated changing color parameters across a single time frame, either after 1 h or 24 h, but not both [4,44]. Vast color changes occurring between T1 and T2 may result from increasing enzymatic activity levels, and increased total phenolic concentration detected post-harvest across cultivars (Figure 2A,B) [47]. This general trend of elevated PPO and TPC levels in apples leading to enzymatic browning has been demonstrated extensively [4,14]. Although, weak correlations were recorded between enzyme activity and color change (PPO:ΔE, *R*^2^ = 0.32; POD:ΔE, *R*^2^ = −0.11) in Persic et al. [44], along with no correlation between TPC:ΔE (*R*^2^ = 0.06), suggesting that there may be other mechanisms involved with flesh oxidation and color changes. Tang et al. [13] recently demonstrated browning unrelated to PPO activity in ‘Fuji’, highlighting alternative oxidative pathways involved in apple browning.

### 3.2. Enzyme Activity Varies across Time and Cultivar

Peroxidase (POD) and polyphenol oxidase (PPO) activities were evaluated across time points and cultivars. Average POD activity levels across all cultivars remained stable between T0 and T1, although it increased significantly (41%) between T1 and T2 (Figure 2A). A similar trend was noted for PPO, with 47% higher levels detected across cultivars at T2 than T0 (Figure 2A). POD and IAAox, an additional oxidative enzyme, demonstrated increased activity in apple fruit ripening [41]. IAAox, which was evaluated at two different absorbances (247 and 254 nm), shows minor increases in activity across time, although not statistically significant (Figure 2B). IAAox has been related to fruit senescence, ripening, and decay in apples. In contrast, the plant hormone indole-3-acetic acid (IAA) has demonstrated the ability to prevent fruit-drop, halt ripening, and improve post-harvest pathogenic control [41,48]. PPO and POD were only evaluated at a single time point in previous studies [4,44]. However, these enzyme levels were significantly higher in the apple peel than the flesh (which was the study’s tissue of focus). As these oxidative enzymes are located in plastids, it is expected that the exterior of the fruit would have a much higher concentration of these “pigment-containing” organelles [13].

**Figure 3:**
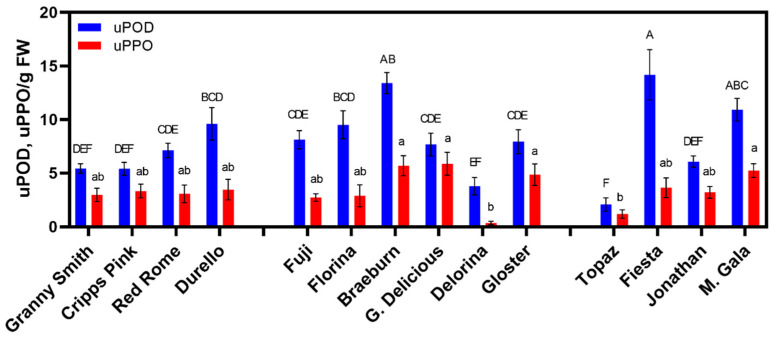
Average enzyme activity for POD (uPOD) and PPO (uPPO). Means displayed are averages of all three times. Means ± standard error displayed. Different letters above bars indicate differences according to Student-Newman-Keuls (SNK) post-hoc mean comparison at a *p* < 0.05. No significant interactions were detected by cultivar x time for enzymatic activities when assessed by ANOVA at *p* < 0.05.

POD and PPO activity showed significant variability across the 14 cultivars regardless of sampling time (Figure 3). Similarly, variable enzymatic activity was previously documented across several apple varieties [32]. Elevated levels of POD activity were pronounced in ‘Fiesta’ and ‘Mondial Gala’, two “severe browning” varieties, along with ‘Braeburn’, a “moderate browning” variety (Figure 3). The lowest POD activity levels were detected in ‘Granny Smith’ and ‘Cripps Pink’, two of the “minimal browning” varieties, along with ‘Delorina’, a “moderate browning” variety. Interestingly, the lowest POD activity levels were in ‘Topaz’, a variety that experienced extreme color changes, especially at T2 (Figure 3). Another severe color-changing cultivar at T2, ‘Jonathan’, also had lower POD levels (Figure 3). High POD and low PPO activity were noted in ‘Florina’ in the present study (Figure 3), which was similarly demonstrated in Persic et al. [44]. A similar observation was also noted previously, where ‘Florina’ had low PPO activity and browned minimally [49]. The high levels of POD in ‘Florina’ in the present study may be why it browned semi-moderately post-harvest, even with PPO’s low levels (Figure 3).

In respect to PPO activity, ‘Mondial Gala’ and ‘Braeburn’ were amongst the highest across the cultivars, similar to the POD results (Figure 3). Overall, ‘Golden Delicious’ had the highest PPO levels across all varieties (Figure 3). This contrasts with Persic et al. [44], which demonstrated low PPO and POD activity in ‘Golden Delicious.’ Relatively low levels of PPO in ‘Granny Smith’ and ‘Cripps Pink’ may be the reason why those two cultivars did not report a significant change in flesh color over time in the present study. Persic et al. [44] demonstrated high TPC, high PPO, and high browning in ‘Granny Smith.’ In contrast, high PPO followed by minimal browning was observed in the same cultivar by Amiot et al. [49]. The role of PPO activity in the browning of particular varieties reveals inconsistent results and can be dependent upon the level of maturity and growing region [22]. This may especially be the case in this study, as ‘Granny Smith’ did demonstrate less ripe levels at harvest (data not shown), along with minimal browning (Figure 1).

‘Delorina’ and ‘Topaz’ highlighted similar results for PPO activity and POD activity with the lowest levels overall, despite their more moderate/severe browning classifications (Figure 1 and Figure 3). ‘Topaz’ with low PPO levels along with moderate browning at T1 confirmed results published in the literature [44]. ‘Topaz’ also demonstrated low levels of PPO in Kołodziejczyk et al. [46]. A moderate relationship between POD and PPO activity was noted across varieties in the present study, when outliers were removed (*R*^2^ = 0.65) (data not shown). This trend underscores their similar behavior as defensive mechanisms [50].

IAAox activity did not reveal significant differences across the cultivars evaluated (Figure S1); although some varieties displayed high levels overall, the variability was too large in some cases. Both ‘Topaz’ and ‘Jonathan’ demonstrated vast color changes (i.e., severe browning, especially at T2), but low levels of both PPO and POD, especially in ‘Topaz’ (Figure 1 and Figure 3). However, it is worth noting that these two varieties exhibited the highest IAAox activity levels at the 254 nm absorbance (Figure S1). Furthermore, a moderate relationship between ΔE2 (T2–T1) and IAAox (254 nm) was noted when outliers were removed (*R*^2^ = 0.54, data not shown), perhaps suggesting an additional oxidative pathway that contributes to browning, especially later in post-harvest (24 h post-slicing).

**Figure 4:**
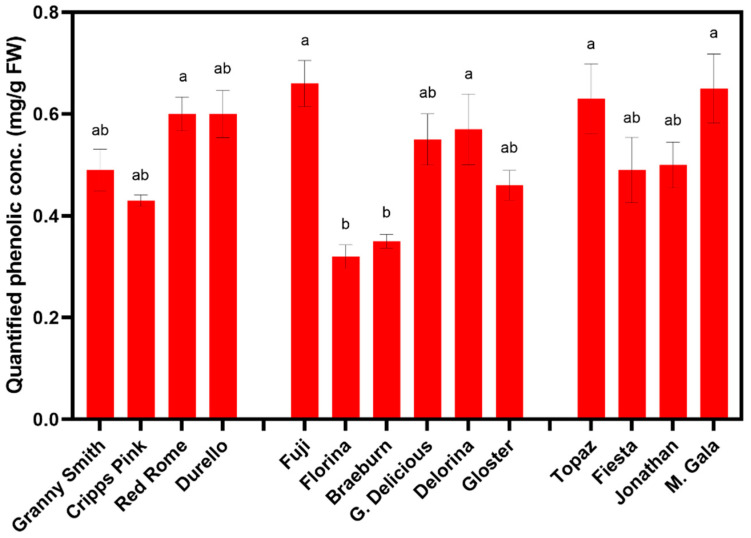
Average quantified phenolic concentration (QPC) per cultivar. Means displayed are averages of all three times. Means ± standard error displayed. Different letters above bars indicate differences according to Student-Newman-Keuls (SNK) post-hoc mean comparison at a *p* < 0.05. No significant interaction was detected by cultivar x time for quantified phenolic concentration when assessed by ANOVA at *p* < 0.05.

Additionally, similarities have been previously noted between ‘Topaz’ and ‘Jonagold’, a progeny of ‘Jonathan’, each containing high levels of quercetin glycosides, a flavanol primarily found in the skin, but also in the flesh, and may contribute to browning [51,52]. In the present study, ‘Topaz’ demonstrated high QPC levels, and so perhaps even with low levels of oxidative enzymes such as PPO and POD, severe browning can still occur (Figure 4). This may be due to the vast abundance of the substrate or the distinct composition of the phenolic compounds present in this cultivar.

### 3.3. Phenolic Compound Concentration Influenced by Time and Apple Cultivar

When assessing the composition of phenolic compounds in the apple varieties, HPLC analyses confirmed the presence of catechin, chlorogenic acid, epicatechin, *p*-coumaric acid, phloridzin, and proanthocyanidins (Table 1 and Table 2). These compounds’ presence is affirmed by previous literature [32,45] and contributes to apples’ antioxidant activity [53,54]. Quantified phenolic concentration (QPC) was determined by summing all the phenolic compounds detected (Table 1 and Table 2) and are displayed in Figure 4 (time points averaged).

On average, QPC increased over time with an 18% increase from T0 to T2, while QPC remained relatively stable between T0 and T1 (Table 1). When evaluating the individual phenolic compounds, only epicatechin displayed a significant increase in concentration over time, from T0 to T2 (Table 1). The other compounds generally increased over time, contributing to QPC’s significant increase from T0 to T2, but none large enough to denote a significant increase between time points at *p* < 0.05 (Table 1). Overall, when evaluating QPC changes over time, they appear to be primarily a result of shifting proanthocyanidin concentrations, as this flavanoid class seems to be the most abundant across all cultivars (Table 1 and Table 2 and Table S1 and Figure 2). Proanthocyanidins (which can include monomeric flavan-3-ols, along with oligo- and polymeric procyanidins) have been confirmed to be the dominant phenolic class in apples throughout the literature [52,53,54].

Catechin has been shown to be an excellent substrate for PPO [32], although, in the present study, its concentration did not result in statistical differences between time points (Table 1). This may be the reason why the cultivars showing minimal browning maintain stable catechin concentration over time (e.g., ‘Cripps Pink’ and ‘Granny Smith’; Tables S1 and S2). PPO activity and enzymatic browning appear to depend on the degree of polymerization of phenolic compounds used as substrates [55]. In other words, smaller monomeric compounds with lower molecular weight, such as catechin and hydroxycinnamic acids (e.g., *p*-coumaric acid and chlorogenic acid), appear to serve as a more efficient substrate for PPO [2,35]. This may explain why varieties such as ‘Granny Smith’ and ‘Cripps Pink’ which demonstrated the lowest levels of these efficient substrates for PPO, such as *p*-coumaric acid, catechin, and chlorogenic acid, browned the least (Table 2). Furthermore, these two varieties did not show differences in QPC compared to the other varieties (Figure 4). Thus, in-depth characterization and understanding of the polymerization of the phenolic compound composition is required, rather than QPC alone, to investigate the phenomena of browning more effectively.

When times were averaged, ‘Florina’ still demonstrated the lowest QPC (Figure 4 and Table 2). The other variety presenting the lowest QPC overall was ‘Braeburn’ (Figure 4 and Table 2), which was also noted by Vanzani et al. [53]. However, ‘Braeburn’ exhibited the highest *p*-coumaric acid levels (Table 2), an efficient substrate for PPO, and may explain its “early” browning between T1 and T0. The highest QPC values were detected in ‘Fuji’, ‘Mondial Gala’, ‘Topaz’, and ‘Red Rome’ (Figure 4 and Table 2).

**Figure 5:**
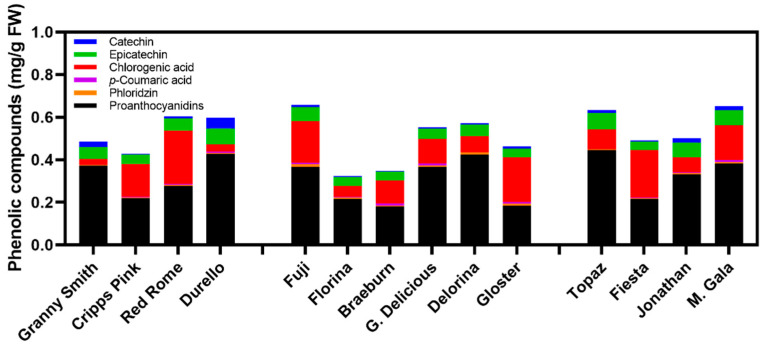
Composition of the phenolic compounds evaluated via high-performance liquid chromatography (HPLC) organized by each phenolic compound class in each cultivar (averaged from all times). Cultivars are presented in the same order as in Figure 1 based on their level of flesh browning from minimal to severe. Means are displayed for each compound within each cultivar column bar. Statistics available for each compound are available in Table 2.

These four varieties that presented high QPC levels also reflect higher levels of proanthocyanidins (Figure 5). This was mostly the case with ‘Topaz’, as it contains the highest abundance of proanthocyanidins amongst all cultivars evaluated (Table 2 and Figure 5). Overall, proanthocyanidins contributed significantly to the QPC of most varieties, demonstrating a robust linear relationship (*R*^2^ = 0.73) between these two factors (data not shown). Vrhovsek et al. [30] also showed that flavanols (sum of catechin and proanthocyanidins) comprised 71% to 90% of TPC across eight apple varieties. Flavanols in this study (epicatechin, catechin, and proanthocyanidins) totaled 93% of QPC in ‘Granny Smith’ (highest across all varieties; data not shown), which was similar to the 90% in Vrhovsek et al. [30]. This high abundance of flavanols, especially proanthocyanidins (Figure 5), may be the reason why ‘Granny Smith’ minimally browns, as proanthocyanidins have demonstrated strong inhibition of PPO activity [35,46]. Conversely, ‘Braeburn’, which browns moderately, had the lowest procyanidins levels (Table 2 and Figure 5). ‘Braeburn’s combination of high PPO, but low QPC, comprised of a high abundance of *p*-coumaric acid and lacking high levels of proanthocyanidins, may help illuminate the complexity involved in its’ enzymatic browning behavior (Table 2 and Figure 1, Figure 3 and Figure 4). Again, Persic et al. [44] noted negligible correlations between PPO:ΔE (*R*^2^ = 0.32) and TPC:ΔE (*R*^2^ = 0.06), highlighting the vast dynamics involved in browning and the other potential mechanisms that contribute to enzymatic oxidation [13]. Furthermore, Persic et al. [44] demonstrated a moderate relationship between PPO:TPC (*R*^2^ = 0.53), while Kołodziejczyk et al. [46] concluded with no correlation between PPO:TPC (*R*^2^ < 0.40) at all across 22 apple cultivars. Overall, quantification of enzyme activity and phenolic concentration is not sufficient to predict browning. Instead, it is a comprehensive understanding of the phenolic compounds’ composition, as phenolic compounds are strong “manipulators” of enzymatic activity, whether inhibition or promotion [2,34].

Two varieties further underscore the complexity of oxidative enzymatic browning in this present study: ‘Topaz’ and ‘Red Rome’.

‘Topaz’ demonstrates the highest levels of proanthocyanidins amongst varieties (Table 2 and Figure 5) along with the lowest levels of PPO and POD (Figure 3) [46], further confirming the role of these highly polymerized compounds’ in inhibiting enzymatic activity [35]. However, between T1 and T2, ‘Topaz’ dramatically changed color (Figure 1). Although PPO and POD activity increased during this time (data not shown), so did the abundance of proanthocyanidins (Tables S1 and S2), suggesting that enzymatic browning should have continued to be inhibited. Nevertheless, a color change occurred dramatically during this 24 h period (T2–T1), perhaps suggesting alternative oxidation pathways [13,44]. Furthermore, this alternative oxidation pathway may be explained by the high levels of IAAox (at 254 nm) in both ‘Topaz’ and ‘Jonathan’ (Figure S1).

‘Red Rome’ is characterized by an alternative narrative with similarly high levels of QPC as ‘Topaz’, but with moderate levels of proanthocyanidins, PPO, and POD (Figure 3 and Figure 4, Table 2). High levels of TPC in ‘Red Rome’ have been confirmed previously [45]. Interestingly, with respect to the phenolic composition, ‘Red Rome’ demonstrated the highest chlorogenic acid concentrations, an excellent PPO substrate [46], and yet the variety browned minimally. Given the high levels of QPC (especially chlorogenic acid) and moderate levels of PPO and POD, without the inhibition of abundant procyanidins, ‘Red Rome’ would be hypothesized to brown, and yet it only minimally changed color (Figure 1). The minimal browning may result from its less mature status at harvest compared to the other varieties (data not shown).

In sum, these exceptional cases of why ‘Topaz’ changed color dramatically, while ‘Red Rome’ changed minimally, given their phenolic compositions, enzymatic activities, and maturity status continue to underscore the narrative of a complex series of biochemical reactions that contribute to enzymatic browning in apples [13,22].

### 3.4. Principal Component Analysis Reveals Cultivar “Clusters” Based on Biochemical Characteristics and Browning Behaviors

**Figure 6:**
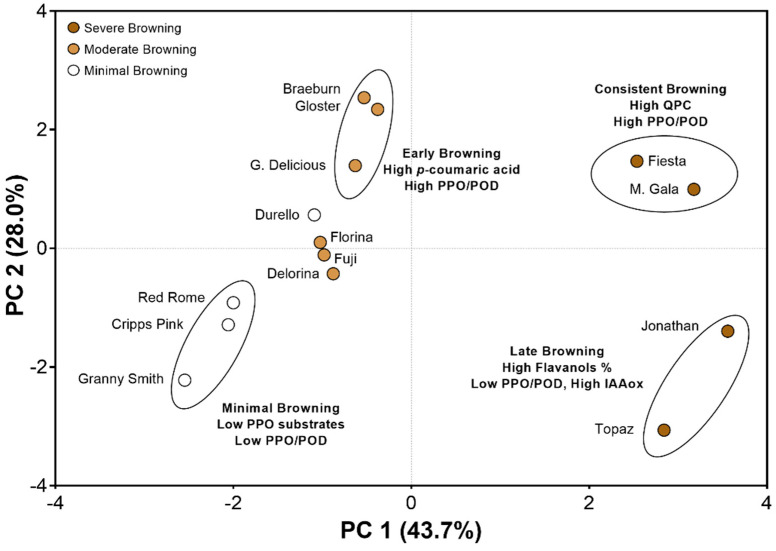
Principal component analysis (PCA) of apple cultivar characteristics such as color changes (i.e., browning), phenolic concentrations, and enzymatic activity. Large circles (scores) visualize the 14 cultivars, colored according to their browning severity (white = minimal, light brown = moderate, and dark brown = severe). PCA showcases that browning coloration and phenolic concentrations were a major contributor to variation amongst cultivars, with ~44% of the variation explained on PC1. An additional variation is explained along PC2 (28%) with enzymatic activity (PPO and POD) appearing to drive vertical separation amongst cultivars evaluated.

A principal component analysis (PCA) was conducted considering each cultivar’s biochemical, enzymatic, and color-changing parameters. This analysis included: QPC, ΔE1, ΔE2, ΔC*1, ΔC*2, PPO, POD, and IAAox (at 254 nm). Based on these factors, PC1 showcased nearly 44% of the variation, while PC2 described 28%, for a total of 72% of all variation explained by these factors across the cultivars evaluated (Figure 6). The varieties were color-coded in Figure 6 according to their color-changing severity. However, they appeared to cluster with other varieties that behaved similarly regarding the timing of coloration, enzymatic activity, and phenolic composition.

Minimal browning varieties presented low enzyme activity and a low abundance of monomeric substrates for PPO such as catechin, chlorogenic acid (except for ‘Red Rome’), and *p*-coumaric acid (Figure 6). Moderate browning varieties that exhibited “early browning” (between T0 and T1) included ‘Braeburn’, ‘Gloster’, and ‘Golden Delicious’, and were characterized by high levels of *p*-coumaric acid, a hydroxycinnamic acid, and efficient PPO substrate [2], along with high PPO activity (Figure 6). High levels of hydroxycinnamic acids have been detected in ‘Golden Delicious’ previously [29,54]. Other minimal and moderate browning varieties such as ‘Durello’, ‘Florina’, ‘Fuji’, and ‘Delorina’ did not reveal clear discrimination between the abovementioned clusters, although they appear to cluster together. ‘Florina’ had the lowest QPC amongst varieties, while ‘Delorina’ had the lowest PPO (Figure 3 and Figure 4).

Lastly, the severe browning varieties appear to cluster and behave in two distinct groups/patterns, underscoring this process’s complexity (Figure 6). All of these varieties demonstrated high QPC (Figure 4), but ‘Fiesta’ and ‘Mondial Gala’ revealed higher enzymatic activity, notably ‘Fiesta’ with POD and ‘Mondial Gala’ with PPO and POD (Figure 3). This may be why they showed consistent and high levels of browning at T1 and T2 (Figure 1 and Figure 6), mainly since ‘Fiesta’ contains low amounts of PPO inhibitory proanthocyanidins (Table 2) [35]. ‘Topaz’ and ‘Jonathan’ appeared to cluster together based on low PPO/POD activity and high QPC, with a high percentage attributed to flavanols (Figure 5 and Figure 6). The enzyme activity levels and inhibitory proanthocyanidins would suggest minimal browning, but they change color dramatically between T1 and T2 (i.e., “late browning;” Figure 1 and Figure 6). This may be due to the higher levels of IAAox within these cultivars (Figure S1), as this additional oxidative enzyme has previously demonstrated a role in fruit senescence and decay [41]. Alternatively, the browning severity may be due to QPC’s exceptional levels still triggering enzymatic browning even when PPO/POD levels are reduced. Severe browning in these varieties may also suggest additional oxidative pathways that may require further investigation [44], such as unrelated PPO browning and the activity of antioxidant enzymes like superoxide dismutase (SOD) in Tang et al. [13]. Overall, although enzymatic browning appears to be a highly complex process, the PCA appears to sufficiently explain similar cultivars’ behaviors clustering based on biochemical composition, enzymatic activities, and color changes.

## 4. Conclusions

The presence of the major phenolic compounds such as catechin, epicatechin, *p*-coumaric acid, chlorogenic acid, and proanthocyanidins are confirmed in these 14 apple cultivars. These phenolic compounds act as enzymatic modifiers, both as suppressors (e.g., proanthocyanidins) and promoters (e.g., monomeric catechin and hydroxycinnamic acids). We noticed that polyphenol oxidase (PPO) and peroxidase (POD) increased in activity across the time post-harvest, as well as quantified phenolic concentration (QPC). Generally, cultivars with high QPC and high enzymatic activity led to more moderate or severe browning. Although oxidative browning appears to be more complicated than this simple formula, QPC’s composition seems to play an important role. In particular, the polymerization of individual phenolic compounds influences enzymatic activity differently. Furthermore, additional oxidative pathways may facilitate browning in apples, as some varieties such as ‘Topaz’ demonstrate high QPC, low PPO, and yet still brown severely after 24 h.

## Figures and Tables

**Table 1:** Phenolic compound concentrations at each time point after cutting (averaged from all cultivars) and quantified phenolic concentration (QPC).

Time	Catechin (mg/g FW)	Epicatechin (mg/g FW)	Chlorogenic Acid (mg/g FW)	*p*-Coumaric Acid (mg/g FW)	Phloridzin (mg/g FW)	Proanthocyanidins (mg/g FW)	Quant. Phenolic Conc. (mg/g FW)
T0	0.014	0.05 b	0.11	0.005	0.005	0.30	0.49 b
T1	0.010	0.05 b	0.12	0.005	0.006	0.28	0.47 b
T2	0.016	0.07 a	0.15	0.005	0.007	0.36	0.60 a
LSD	nd	0.02	nd	nd	nd	nd	0.11

LSD = least significant difference as determined by Tukey HSD *p* < 0.05; “nd” indicates no difference at *p* < 0.05; Means with the same letter indicate non-significance at a *p* < 0.05.

**Table 2:** Phenolic compound concentrations within each cultivar (averaged from all time points) and quantified phenolic concentration (QPC). Cultivars are presented in the same order as in Figure 1 based on their level of flesh browning: minimal browning (first 4 cultivars), moderate browning (the portion of table shaded in gray), and severe browning (last 4 cultivars).

Cultivar	Catechin (mg/g FW)	Epicatechin (mg/g FW)	Chlorogenic Acid (mg/g FW)	*p*-Coumaric Acid (mg/g FW)	Phloridzin (mg/g FW)	Proanthocyanidis (mg/g FW)	Quant. phenolic Conc. (mg/g FW)
‘Granny Smith’	0.026 b	0.06 ab	**0.03 f**	**0.002 c**	0.004 cd	0.37 abc	0.49 abc
‘Cripps Pink’	**0.003 d**	0.04 b	0.16 bcd	0.003 c	0.004 d	0.22 bc	0.43 abc
‘Red Rome’	0.010 cd	0.06 ab	**0.25 a**	0.005 bc	0.005 bcd	0.28 abc	0.60 a
‘Durello’	**0.049 a**	0.08 a	0.03 ef	0.006 ab	0.006 abcd	0.43 a	0.60 ab
‘Fuji’	0.010 cd	0.07 ab	0.20 abc	0.006 abc	**0.014 a**	0.37 abc	**0.66 a**
‘Florina’	0.005 cd	0.04 b	0.05 ef	0.001 d	0.006 abc	0.22 bc	**0.32 c**
‘Braeburn’	0.003 d	0.04 b	0.11 def	**0.011 a**	0.005 abcd	**0.18 c**	0.35 bc
‘G. Delicious’	0.004 d	0.05 ab	0.12 cde	0.008 a	0.008 ab	0.37 abc	0.55 abc
‘Delorina’	0.007 cd	0.05 ab	0.08 def	**-**	0.011 a	0.42 a	0.57 abc
‘Gloster’	0.011 bcd	**0.04 b**	0.21 ab	0.008 ab	0.009 ab	0.18 c	0.46 abc
‘Topaz’	0.013 bcd	**0.08 a**	0.09 def	**-**	**0.003 d**	**0.45 a**	0.63 a
‘Fiesta’	0.005 d	0.04 b	0.22 ab	0.003 c	0.003 bcd	0.22 bc	0.49 abc
‘Jonathan’	0.019 bc	0.07 ab	0.07 ef	0.005 bc	0.005 abcd	0.33 abc	0.50 abc
‘M. Gala’	0.020 bcd	0.07 ab	0.16 bcd	0.010 bc	0.006 abcd	0.38 ab	0.65 a
LSD	0.015	0.03	0.09	0.005	0.007	0.20	0.26

In each column, bold highlights the highest and lowest values in each column; LSD = least significant difference as determined by Tukey HSD at *p* < 0.05; Means with the different letters (on the right side of each value) within each column indicate significant differences at *p* < 0.05.

## Data Availability

The data presented in this study are available in here and in supplementary material.

## Supplementary Materials

The following are available online at https://www.mdpi.com/2304-8158/10/1/186/s1. Figure S1: Average enzyme activity for IAAox (in IAAox units/g FW) at two different wavelengths (247 nm and 254 nm) for each cultivar. The means displayed are averages from all three times. Means ± standard error displayed. No significant differences were detected across cultivars according to Tukey’s HSD post-hoc mean comparison at a *p* < 0.05., Table S1: Phenolic compound concentrations within each cultivar at each time point post-slicing (T0, T1, T2). Cultivars are presented in the same order as in Figure 1 based on their level of flesh browning: minimal browning (first 4 cultivars), moderate browning (portion of table shaded in gray), and severe browning (last 4 cultivars), Table S2: The standard deviation of phenolic compound concentrations to associate to the corresponding mean value in Table S1 within each cultivar at each time point post-slicing (T0, T1, T2). Cultivars are presented in the same order as in Figure 1 based on their level of flesh browning: minimal browning (first 4 cultivars), moderate browning (portion of table shaded in gray), and severe browning (last 4 cultivars).
