# Supplementary material for: Determination of Post-Harvest Biochemical Composition, Enzymatic Activities, and Oxidative Browning in 14 Apple Cultivars
Source: Foods. 2021 Jan 18;10(1):186. doi: 10.3390/foods10010186 (PMC7831482; doi:10.3390/foods10010186)
Supplement: Supplementary file 1 [file foods-10-00186-s001.pdf]

Supplementary Materials:

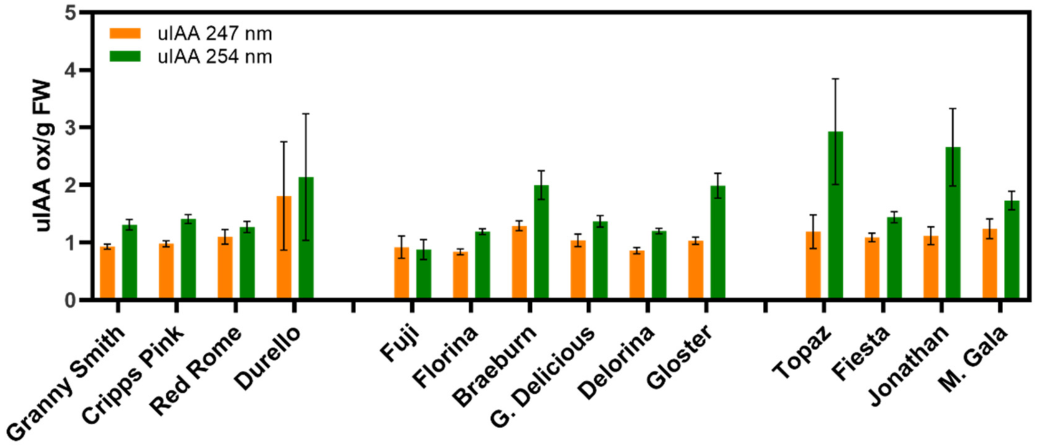

Figure S1. Average enzyme activity for IAAox (in IAAox units/g FW) at two different wavelengths (247 nm and 254 nm) for each cultivar. The means displayed are averages from all three times. Means ± standard error displayed. No significant differences were detected across cultivars according to Tukey's HSD post-hoc mean comparison at $p < 0.05$.

Table S1. Phenolic compound concentrations within each cultivar at each time point post-slicing (T0, T1, T2). Cultivars are presented in the same order as in Figure 1 based on their level of flesh browning: minimal browning (first 4 cultivars), moderate browning (portion of table shaded in gray), and severe browning (last 4 cultivars).

	**Catechin** (mg/g FW)			**Epicatechin** (mg/g FW)			**Chlorogenic acid** (mg/g FW)			***p*-Coumaric acid** (mg/g FW)			**Phloridzin** (mg/g FW)			**Proanthocyanidins** (mg/g FW)		
**Cultivar**	**T0**	**T1**	**T2**	**T0**	**T1**	**T2**	**T0**	**T1**	**T2**	**T0**	**T1**	**T2**	**T0**	**T1**	**T2**	**T0**	**T1**	**T2**
'G. Smith'	0.026	0.026	0.025	0.03	0.07	0.07	**0.02**	**0.03**	**0.04**	0.002	**0.002**	0.002	0.004	0.004	0.005	0.35	0.42	0.34
'Cripps Pink'	0.003	0.003	0.004	0.05	0.04	0.05	0.14	0.14	0.18	0.002	0.002	0.003	0.003	0.004	0.003	0.21	0.22	0.23
'Red Rome'	0.012	0.007	0.012	0.05	0.05	0.06	**0.23**	**0.23**	**0.30**	0.004	0.005	0.006	0.004	0.005	0.005	0.26	0.28	0.29
'Durello'	**0.057**	**0.040**	**0.050**	0.07	**0.07**	0.09	0.03	0.03	0.04	0.006	0.005	0.007	0.005	0.005	0.007	0.37	**0.46**	0.45
'Fuji'	0.011	0.012	0.008	0.06	0.05	0.08	0.21	0.17	0.21	0.005	0.005	0.007	**0.012**	0.010	**0.019**	0.37	0.29	0.44
'Florina'	0.004	0.007	0.006	0.04	0.04	**0.04**	0.04	0.04	0.07	**0.001**	**-**	**0.002**	0.005	0.006	0.008	0.24	0.18	0.22
'Braeburn'	**0.003**	0.004	**0.003**	0.04	0.04	0.05	0.09	0.10	0.13	**0.010**	0.011	0.011	0.004	0.005	0.006	0.18	**0.16**	**0.20**
'G. Delicious'	0.004	-	0.008	0.05	0.04	0.06	0.10	0.11	0.13	0.008	0.008	0.008	0.006	0.008	0.009	**0.44**	0.24	0.42
'Delorina'	0.009	**0.002**	0.010	0.05	0.05	0.06	0.06	0.09	0.08	-	-	-	0.009	**0.013**	0.012	0.41	0.44	0.42
'Gloster'	0.009	0.006	0.019	0.04	**0.03**	0.05	0.17	0.20	0.26	0.009	0.006	0.010	0.007	0.009	0.010	0.19	0.15	0.21
'Topaz'	0.017	0.006	0.015	**0.07**	0.07	**0.10**	0.08	0.10	0.10	-	-	-	0.003	0.003	**0.003**	0.41	0.30	**0.62**
'Fiesta'	-	**-**	0.015	**0.03**	0.04	0.05	0.20	0.21	0.26	0.003	0.003	0.003	**0.002**	**0.003**	0.003	**0.14**	0.19	0.32
'Jonathan'	0.020	0.013	0.024	0.06	0.06	0.08	0.06	0.07	0.08	0.005	0.004	0.005	0.005	0.004	0.005	0.30	0.25	0.44
'M. Gala'	0.020	0.014	0.026	0.06	0.07	0.08	0.16	0.16	0.17	0.009	**0.011**	**0.011**	0.005	0.007	0.005	0.39	0.30	0.45
LSD		0.035			0.06			0.21			nd			nd			0.41	

In each column, bold highlights the highest and lowest values in each column. All means (cultivar × time (T0, T1, T2)) for each phenolic compound compared by Tukey HSD at $p < 0.05$. LSD = least significant difference as determined by Tukey HSD; means with values larger than LSD indicates significance at $p < 0.05$; nd = no difference at $p < 0.05$.

Table S2. The standard deviation of phenolic compound concentrations to associate to the corresponding mean value in Table S1 within each cultivar at each time point post-slicing (T0, T1, T2). Cultivars are presented in the same order as in Figure 1 based on their level of flesh browning: minimal browning (first 4 cultivars), moderate browning (portion of table shaded in gray), and severe browning (last 4 cultivars).

	**Catechin** (mg/g FW)			**Epicatechin** (mg/g FW)			**Chlorogenic acid** (mg/g FW)			***p*-Coumaric acid** (mg/g FW)			**Phloridzin** (mg/g FW)			**Proanthocyanidins** (mg/g FW)		
**Cultivar**	**T0**	**T1**	**T2**	**T0**	**T1**	**T2**	**T0**	**T1**	**T2**	**T0**	**T1**	**T2**	**T0**	**T1**	**T2**	**T0**	**T1**	**T2**
'G. Smith'	0.010	0.002	0.001	0.033	0.011	0.022	0.008	0.021	0.000	0.000	0.000	0.000	0.001	0.000	0.000	0.163	0.034	0.078
'Cripps Pink'	0.004	0.004	0.006	0.002	0.006	0.013	0.040	0.057	0.063	0.000	0.000	0.000	0.000	0.001	0.001	0.045	0.014	0.019
'Red Rome'	0.000	0.010	0.002	0.006	0.002	0.012	0.017	0.027	0.069	0.000	0.000	0.002	0.000	0.001	0.001	0.059	0.063	0.057
'Durello'	0.024	0.005	0.016	0.021	0.015	0.006	0.008	0.019	0.007	0.000	0.002	0.002	0.001	0.001	0.000	0.103	0.143	0.045
'Fuji'	0.016	0.004	0.012	0.009	0.017	0.035	0.028	0.052	0.020	0.001	0.002	0.001	0.004	0.001	0.007	0.037	0.057	0.007
'Florina'	0.005	0.002	0.009	0.005	0.017	0.004	0.025	0.009	0.072	0.001	0.001	0.002	0.001	0.001	0.001	0.043	0.055	0.007
'Braeburn'	0.004	0.006	0.004	0.010	0.002	0.015	0.001	0.026	0.006	0.001	0.002	0.000	0.000	0.001	0.000	0.001	0.028	0.019
'G. Delicious'	0.005	0.000	0.000	0.003	0.005	0.007	0.045	0.047	0.061	0.000	0.002	0.001	0.001	0.001	0.001	0.087	0.021	0.065
'Delorina'	0.002	0.003	0.003	0.003	0.008	0.017	0.006	0.011	0.008	0.000	0.000	0.000	0.002	0.003	0.001	0.108	0.303	0.112
'Gloster'	0.013	0.009	0.001	0.001	0.000	0.001	0.028	0.044	0.007	0.002	0.003	0.001	0.001	0.000	0.001	0.025	0.030	0.044
'Topaz'	0.010	0.008	0.000	0.001	0.004	0.011	0.026	0.057	0.042	0.000	0.000	0.000	0.000	0.002	0.001	0.019	0.092	0.031
'Fiesta'	0.000	0.000	0.002	0.004	0.009	0.004	0.091	0.119	0.152	0.001	0.002	0.003	0.003	0.005	0.005	0.076	0.114	0.188
'Jonathan'	0.004	0.004	0.003	0.003	0.014	0.008	0.014	0.029	0.026	0.001	0.000	0.001	0.001	0.000	0.001	0.003	0.056	0.059
'M. Gala'	0.006	0.000	0.006	0.013	0.024	0.013	0.021	0.030	0.069	0.000	0.003	0.003	0.004	0.003	0.003	0.268	0.121	0.089

Standard deviation displayed for each cultivar × time (T0, T1, T2) for each phenolic compound.
